# Closed-Loop Lifecycle Management of Service and Product in the Internet of Things: Semantic Framework for Knowledge Integration

**DOI:** 10.3390/s16071053

**Published:** 2016-07-08

**Authors:** Min-Jung Yoo, Clément Grozel, Dimitris Kiritsis

**Affiliations:** Swiss Federal Institute of Technology, Mechanical Engineering, ICT for Sustainable Manufacturing, EPFL, CH-1015 Lausanne, Switzerland; clement.grozel@epfl.ch (C.G.); dimitris.kiritsis@epfl.ch (D.K.)

**Keywords:** Closed-Loop Lifecycle Management (CL2M), Internet of Things (IoT), Product Lifecycle Management (PLM), Product-Service ontology, O-DF, O-MI, electric car

## Abstract

This paper describes our conceptual framework of closed-loop lifecycle information sharing for product-service in the Internet of Things (IoT). The framework is based on the ontology model of product-service and a type of IoT message standard, Open Messaging Interface (O-MI) and Open Data Format (O-DF), which ensures data communication. (1) Background: Based on an existing product lifecycle management (PLM) methodology, we enhanced the ontology model for the purpose of integrating efficiently the product-service ontology model that was newly developed; (2) Methods: The IoT message transfer layer is vertically integrated into a semantic knowledge framework inside which a Semantic Info-Node Agent (SINA) uses the message format as a common protocol of product-service lifecycle data transfer; (3) Results: The product-service ontology model facilitates information retrieval and knowledge extraction during the product lifecycle, while making more information available for the sake of service business creation. The vertical integration of IoT message transfer, encompassing all semantic layers, helps achieve a more flexible and modular approach to knowledge sharing in an IoT environment; (4) Contribution: A semantic data annotation applied to IoT can contribute to enhancing collected data types, which entails a richer knowledge extraction. The ontology-based PLM model enables as well the horizontal integration of heterogeneous PLM data while breaking traditional vertical information silos; (5) Conclusion: The framework was applied to a fictive case study with an electric car service for the purpose of demonstration. For the purpose of demonstrating the feasibility of the approach, the semantic model is implemented in Sesame APIs, which play the role of an Internet-connected Resource Description Framework (RDF) database.

## 1. Introduction

The Closed-Loop Lifecycle Management (CL2M) of products addresses the ability of collecting useful product information during its full lifecycle phases for the purpose of re-using such information in developing product qualities and enhancing business opportunities [1]. Being applied to the product and service lifecycle management, this issue requires first of all collecting all possible and potentially useful data about products and services for the whole lifecycle. Afterwards, the collected data should be shared and understood interchangeably, from which knowledge can be extracted by combining all gathered information. The knowledge will contribute to defining key product functionalities expected by the end users, or to identifying additional services and functionalities. Therefore, this is an important step to generate added values to products and services. As for the methodology of structuring and managing a wide range of product lifecycle data, a semantic approach based on the ontology model has already been approved as a promising method [2,3].

In today’s innovative environments of Information and Communication Technology (ICT), the data acquisition of Product Lifecycle Management (PLM) calls for open infrastructure, including Internet, cloud computing and big data, with a view toward creating an open ecosystem. Thanks to highly developed sensor technologies, product-related data are being generated from many kinds of sources throughout the lifecycle of products. Data from sensors installed in manufacturing workshops during manufacturing, transactional data during the transportation of finished products, data from warehouse-installed electronic surveillance systems that control the product in-out and product usage data from intelligent products are some such examples. However, for the purpose of taking advantage from the integration of sensor technologies with a semantically-enriched modelling approach, some weaknesses were observed, which need further enhancement. On the one hand, in traditional approaches to sensor data collection and information handling, data from physical products are often generated in a predefined context of a specific object: the integration of semantically annotated data with the other sensor data is still on-going [4]. The approach to a semantic sensor network [5,6] and the achievement seem promising so that the information on the data-providing devices, i.e., sensors, can also be transferred and used for knowledge inference. On the other hand, product data collection is highly restricted to sensor-generated data while excluding, or considering only a limited range of, other types of information on product usage or wear state. Such information can be collected during the products’ Middle of Life (MOL) operations, i.e., during maintenance or repair processes under the form of human technicians’ observation, or service activities operated on products during its usage phase. Even though such data are available, they have been neither systematically collected by manufacturers nor intensively reused for PLM purposes. By strategically closing all types of data while relevantly transforming them into targeted information and knowledge, a manufacturing firm will achieve better business opportunities.

For that purpose, there are still gaps between existing approaches and the expected ideal infrastructure of PLM, which must be narrowed. Some of them have been identified during this research and are summarized into two points: (i) shifting the means of data collection: from achieving exclusively by a dedicated on-product sensor towards an open and IoT-enabled environment; (ii) enlarging the category of data to be collected: integrating not only product-generated data, but also data generated from other sources, such as human observation or user profile data, which help to understand the state of product usage; (iii) applying semantic annotation to an extended range so that information extraction can occur at every level of product life data. 

There are potential business opportunities that manufacturing firms can create thanks to the deployment of extended closed-loop PLM. Here are some examples: 

1. Now, the lifecycle data of a product as-a-whole and its parts can be closed. Thanks to this information closing, the volume of knowledge assets, which are available to and reachable by companies, might be increased and enhanced. This opens up a new horizon of business opportunities since the knowledge assets play an important role in the company’s management and strategic decision for future operations. Such opportunities were underlined as new business challenges in the sector of electric batteries and cars [7]. Here is an example with the electric car/battery presented in Section 4. If a company wants to access the knowledge assets of the previous life history of an electric battery, this might be possible even after that the battery was removed from a car and its usage type has been changed. PLM information will be available at the starting time of the battery’s second life. This enables companies to infer useful knowledge from an extended range of information, while breaking the silos due to their place of creation or initial purpose.

2. It enhances the smooth integration of third-party service providers that offer knowledge-intensive services. Such services can be based on knowledge assets delivered from stakeholders’ PLM information. One such example concerns a recommendation service to customers based on user habits of using products, such as household machines or electric cars. For example, a good battery charging habit is valuable to extending an electric battery’s life span. During the usage phase of the product, such user habits can be traced by third party service providers, whereas other technical specialists or manufacturing firms might provide the necessary technical constraints to be checked.

The main motivation of this research is to propose a conceptual framework of CL2M providing an architectural solution that can improve both drawbacks by: (i) providing a well-defined framework within which Internet of Things (IoT) data can be seamlessly integrated in a semantic ontology layer; (ii) extending an existing ontology of PLM to a larger scale of Product-Service Lifecycle Ontology (PSLO) in order to manage product-service lifecycle data using the framework of knowledge sharing. 

From now on, the paper is organised as follows. In the next section, the state-of-the-art and existing methods are presented in order to define the baseline context of this research. Section 3 discusses the semantic framework for CL2M. In Section 4, the case study example is illustrated which was created as a domain specific instance of the semantic framework. Section 5 deals with some discussions on the approach such as benefits and limitations, which will be followed by conclusions in Section 6. 

## 2. State-of-the-Art and Methods

In this section, we discuss the basic knowledge and research background that supported the work described in this paper. The key questions to answer are listed below:

- What is a Product-Service (PS)?
- Which approaches were already tackled as for the ontology-based PLM?
- Which mechanism of IoT message transfer will best fit our needs?

### 2.1. Product, Service and Product-Service System

In this subsection, we discuss generally accepted definitions of product, service and a Product-Service System upon which the context of this work is based. In the work of [8], the terms related to Product-Service System (PSS) are summarized as follows:

- Product-Service (PS): an integrated combination of tangible products and intangible services.
- Product-Service System (PSS): social systems that enhance social and economic value received by each actor in the network through the mutual provision of a PS.
- Actor: an individual, group or organization that is actively engaged in the PSS (e.g., provider, partner or customer).

In [9], the term PSS has been defined as “a marketable set of products and services capable of jointly fulfilling a user’s need”. The product/service ratio can vary either in terms of function fulfilment or economic value.

The list below outlines various approaches and trends, among many, towards the development of PSS taken from several research works [8,9,10,11,12,13]:

- The sale of the use of the product instead of the product itself.
- The change to a ‘leasing society’.
- The substitution of goods by means of service machines.
- A repair-society instead of a throw-away society.
- The change in consumer attitudes from sales to service orientation.

So far, PSSs are likely to give more attention to the use phase of the product’s life cycle (consumer state) rather than thinking of information reuse within the other phases of lifecycle. The following elements borrowed from [9] characterize the current thinking of PSS.

- Service at the point of sale, comprised of personal assistance in shops, financial schemes, provided to customers, explanations about product use and, of course, marketing.
- The category of product use: use oriented, where the user extracts product utility; and result oriented, where product utility is extracted by the utility provider.
- Maintenance services include servicing of products with the goal of prolonging the product life cycle, comprising maintenance and upgrading.
- The importance of upgrading services aiming at closing the product material cycle by taking products back, secondary utilization of usable parts in new products and recycling of materials if reuse is not feasible.

The last point concerns the end-of-life operation, which is considered as one of the services. The end-of-life service can be achieved as a finalizing operation while playing a key role in closing the material cycles. Closing the information cycles is much more complex and must be followed with care. 

According to [8], the benefits of service offerings are manifold.

- A service offering by manufacturing companies might be a source of continuous revenue as a result of long-term relationships with customers.
- It is a differentiator for competing in global market.
- It gives the capability to offer a flexible value proposition to customers throughout the lifetime of a product.

In [10], the authors discussed the importance of PSS in the context of PSS design and management. They presented a methodology called “smart maintenance service”, which enabled the strategic progress of PSS from product-oriented to a service-oriented lifecycle. They argued that services bring industries competitive advantages from the economic point of view through the cost reduction as well as profit increase.

Several industries, which have already achieved PLM strategies, are still struggling to move to service-centric holistic CL2M. According to [11], the suggestion goes to transfer the manufacturing business strategy from “designing and selling products only” to “support and accompany their usages with services and end-of-life management”. The above-discussed PSS researches are summarized on Table 1.

According to the classification of the business model of product-service [12], such a trend can be regarded as a move from a ‘use-oriented’ towards a ‘result-oriented’ product-service offering. How should the service lifecycle be integrated coherently and seamlessly with legacy PLM data and information? Our experience revealed that the more CL2M data and the environment are heterogeneous, the more a semantic approach turns out to be beneficial. This paper will take a look at the semantic modelling and ontologies in PLM in the following subsection.

### 2.2. Ontology and Semantic Modelling for Product-Lifecycle Management

The question of “what is an ontology?” is very difficult to answer [14] since the answer depends on the objective of the ontology developer with the purpose of ontology use. This subsection starts with giving a generally-accepted definition of ontology and semantic approaches.

Ontology includes a semantic definition. Wikipedia interprets semantics (from Ancient Greek: σημαντικός sēmantikós, “significant”) as the study of meaning. It focuses on the relation between signifiers, like words, phrases, signs and symbols, and what they stand for, their denotation. Linguistic semantics is the study of meaning that is used for understanding human expression through language. Other forms of semantics can include the semantics of programming languages, formal logics and semiotics.

The work in [15] defines the information model and semantic modelling as follows: “An information model provides the ability to abstract different kinds of data and provides an understanding of how the data elements relate. A semantic model is a type of information model that supports the modelling of entities and their relationships”. The total set of entities in our semantic model comprise the taxonomy of classes we use in the model to represent the real world.

Together, these ideas are represented by an ontology: the vocabulary of the semantic model that provides the basis on which user-defined model queries are formed. The model supports the representation of entities and their relationships and can support the constraints on those relationships and entities. This provides the semantic makeup of the information model. It is now generally recognized that an ontology describes the formal constraints of the terms in a common vocabulary and expresses relationships among them, described using a type of ontology representation language. An ontology can describe hierarchical relationships among terms (as in a taxonomy), describe associative relationships among terms (as in a thesaurus) and relate terms within the vocabulary to other terms described outside of the vocabulary.

During a workshop organized by the U.S. National Institute of Standards and Technology (NIST) [14], three common views on ontologies were discussed, which are listed below:

- Ontologies are models of reality: This is the most classical point of view from which the word ‘ontology’ was understood.
- Ontologies are models of information: According to this view, the ontology itself means the information representation of a given domain problem. In this case, it is possible that such an information model is not a model of any reality.
- Ontologies are compendia of controlled terms: In this view, the use of ontology for defining terms and relationships is importantly underlined in order to define the above-mentioned models. The clear definition of relationships makes terms accessible and useful when they are retrieved.

Ontology developers often have one of these uses in mine. Therefore, the developed ontologies are built with particular purposes in mind, which were inadequate in other contexts. By and large, the ontology issues always include the context, i.e., the context in which the utility of the ontology was targeted, the context of the domain that the ontology was intended to model or the context in which the information should be retrieved. Subsequently, without considering such a context, it is hardly possible to figure out for what purpose an ontology was intended.

Ontology models, through their formal semantics, support several useful features, the main ones being: to share common understanding of the structure of information among human or/and software agents; to enable re-use of domain knowledge; to make domain assumptions explicit; to separate domain knowledge from operational knowledge; to provide the formal analysis of terms; and based on them, to analyse the domain knowledge; all were well addressed in [16]. The author assessed as well that formal analysis of terms is extremely valuable when attempting both to re-use and to extend ontologies.

The basic Resource Description Framework (RDF) [17] makes few distinctions in the ways that Uniform Resource Identifiers (URIs) can be used to represent predicates and objects. However, other systems have been developed to extend basic RDF and to provide ways to indicate more clearly the nature of resources and the relationships between them. RDF Schema (RDFS) [18] and Web Ontology Language 2 (OWL2) [19] provide increasingly complex means of describing such relationships.

It is possible to use advanced methods and tools, such as the Semantic Web Rule Language (SWRL), SPARQL - or Simple - Protocol And RDF Query Language (SPARQL) and the Java Expert System Shell (Jess) [20,21,22], to name a few, in order to exploit the inferred knowledge from the model using reasoning capabilities. The use of these methods and tools will support seamless continuation of knowledge so that relevant knowledge should be available whenever necessary.

**Figure 1:**
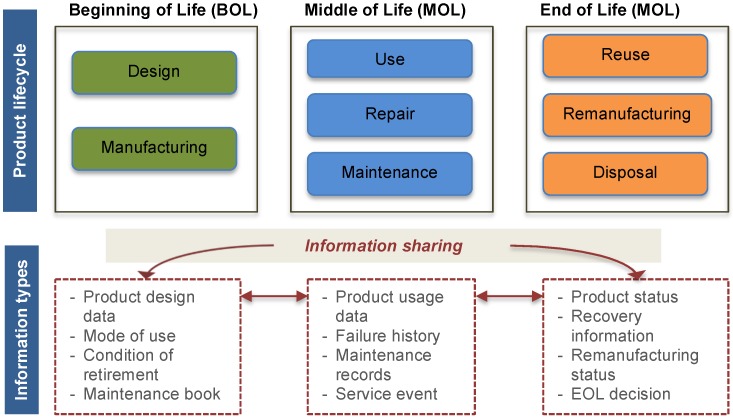
Product lifecycle management and information closing.

The approach to PLM used here is mainly based on the ontological definition and knowledge extraction taking advantage of interoperability, reasoning capability and incremental modelling. In the context of Product Lifecycle Management (PLM), domain professionals have already acknowledged the importance of representing and sharing product data during the different phases of the product lifecycle. There has been significant achievement in integrating product data from Beginning of Life (BOL), to the Middle of Life (MOL), until its End of Life (EOL), especially using shared common ontologies and an intelligent retrieval mechanism [1,2,3,23,24]. Figure 1 illustrates the different PLM phases with the shared information types. PLM has specific objectives in each phase of the lifecycle: during the BOL, the improvement of product design and production quality is the main concern; during the MOL, improving the reliability, availability and maintainability of products are the most interesting issues, for example.

In the context of the work by [2,3], ontology-based closed-loop physical product lifecycle management was addressed. The authors translated, in OWL2, a previously developed Semantic Object Model (PLM SOM), for physical products. The PLM SOM is one of the outcomes from the European project, PROduct lifecycle Management and Information tracking using Smart Embedded Systems (PROMISE) [25]. The initial data were collected from an existing database that contained real-world physical product data. The resulting ontology model is dynamic and incremental.

The relevance of an ontology-based approach to the closed-loop PLM is evident, so much that it has furthered the research interest to the extension of an existing model. The main reasons for reusing PLM SOM as the base of extension are summarized as follows: (i) the model consistency of the existing ontology was already validated; (ii) the instances, which were delivered from real product data, are provided as an initial dataset on which tests of the ontological search could be conducted; (iii) as for the MOL ontology, the physical product data should be semantically coherent with the service life data. The PLM SOM data included automobile parts’ information, which is semantically coherent with the electric car ontology used in this work, even though the data did not directly concern electric car products. Therefore, from the perspectives of data reusability, the extension of PLM SOM has been justified.

### 2.3. Internet of Things and Semantic Modelling Approach

A variety of applications, data sources, exchange formats and transport protocols requires a high level of flexibility and scalability on the IoT. Besides the traditional approaches to data integration, a number of semantic approaches remains to be taken into account. Big data solutions and cloud platforms can provide infrastructure and tools for processing and analysing the huge amount of IoT data. However, efficient methods and solutions are still required that can structure, annotate, share and make sense of the IoT data, which facilitate transforming raw data into actionable knowledge and intelligence in different application scenarios. Experience with the semantic sensor network [5,6] is to be underlined in such perspectives. The advantages of using semantic modelling approaches in the infrastructure of IoT are discussed further by [26], which are summarized below:

1. Semantics for interoperability: This means that different stakeholders can access and interpret the data unambiguously. Things on the IoT need to exchange data with each other and with other users on the Internet. Semantic annotation of the data can provide machine-interpretable descriptions about what the data represent, where they originate from, how they can be related to the surroundings, who is providing them and what are the quality, technical and non-technical attributes.
2. IoT data integration: IoT data usually originate from a device or a human and refer to the attributes of a phenomenon or an entity in the physical world. The data can be combined with other data to create different abstractions of the environment or they can be integrated into the data processing chain in and existing application to support context and situation awareness. Semantic descriptions can support this integration by enabling interoperability between different sources.
3. Resource/service search and discovery: In the IoT, a resource is referred to as a device or entity that can provide data or perform actuation (e.g., a sensor or an actuator), and a service is a software entity that exposes the functionality of its corresponding resource. The search and discovery mechanisms allow locating resources or services that provide data related to an entity of interest in the physical world. Semantically-annotated data and apps can be processed by and retrieved by intelligent reasoning tools, therefore giving the possibility of integrating an e-ecosystem layer for smart search and knowledge retrieval.

The work presented in [27] clearly demonstrates that the sensor systems are taking advantage of semantic annotation and reasoning capability to develop an intelligent problem-solving framework within which the function of matching sensors to compatible algorithms in surveillance systems is effectively integrated.

### 2.4. Open Messaging Interface/Open Data Format: The Open Group IoT Standards for Message Synchronization and Semantic Integration

As far as the standards are concerned, Internet of Things standards, such as O-MI (Open Messaging Interface) [28] and O-DF (Open Data Format) [29], facilitate the collection of information on things into a semantically-provided knowledge structure [30]. O-MI/O-DF, which is The Open Group [31] Internet of Things message standards worked out by the IoT Workgroup [32], emerged from the PROMISE EU FP6 project. Information, such as sensor readings, alarms, manufacturing, disassembly and supply chain events and other information related to the entire product lifecycle, needs to be exchanged between products and systems of different organizations. O-MI/O-DF is one such message interfacing standard that satisfies all of the requirements.

The key idea of O-DF and O-MI is to make available the management of product lifecycle information while tracing how the product has behaved or how the customer has used each individual product. The set of information can be provided through the data generated by a product-instance with the help of sensors and actuators. Thanks to the IoT-related technologies, a very large volume of data, in a wide range, can be collected. However, due to the diversity of information sources, the analysis and knowledge extraction from those data becomes too tricky to cope with.

Here are some functional requirements for an IoT message to be suitable for a PLM context [30]:

- Possible to implement for any kind of instance as independently of the application domain as possible.
- Possible to implement for any kind of information system, including embedded and mobile systems.
- Support for ‘synchronous’ messaging, such as immediate read and write operations, including ‘client-poll’ subscriptions.
- Not restricted to one communication protocol only; it must be possible to send messages using protocols, such as plain HTTP, Simple Object Access Protocol (SOAP), simple Mail Transfer Protocol (SMPT), as file copies, etc.
- Possible to create ad hoc, loosely-coupled tie to limited information flows on the fly.
- Peer-to-peer communication possibility for all devices, i.e., client and server functionality can be implemented for any device, depending on available processing power, network, connectivity, etc.
- Handling mobility and intermittent network connectivity, i.e., support for asynchronous messaging capabilities that imply for instance message persistence, time-to-live, etc.
- Context-dependent discovery of instances, instance-related services and meta-data about them.
- Support for context and domain-specific ontologies.
- Queries by regular expression for retrieving information about more than one instance and more than one kind of information.
- Historical queries, i.e., retrieving values between two points in time.

The last four points are particularly important options to be satisfied so that IoT data can be combined with a semantic modelling approach. Here are some beneficial aspects from applying O-MI/O-DF to ontology-enabled PLM, which were found from our research:

- Support for context and domain-specific ontologies is an important point for the purpose of integrating several domain problems into a global point of view.
- The query possibility is a mandatory factor with the aim of knowledge extraction.
- The possibility of specifying time ranges is useful so that knowledge inference can occur while including temporal constraints. This function helps improve the quality of data search.

**Figure 2:**
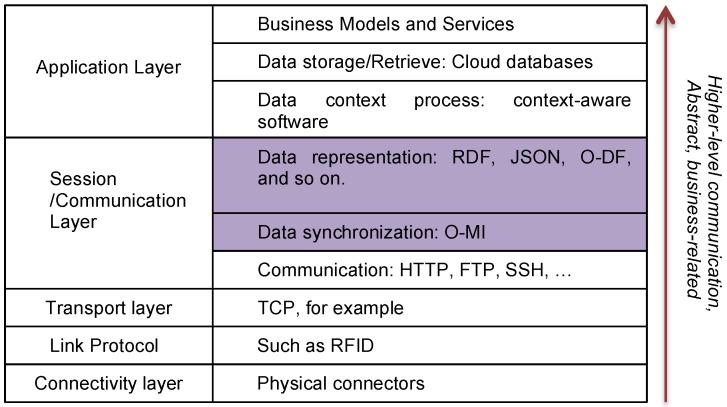
O-MI/O-DF transport layer inside the landscape of IoT data transfer.

O-MI can be used for transporting payloads also in formats other than O-DF (such as RDF data structures). O-DF can be viewed as a generic content description model for things in the IoT. A simple example of an O-MI and an O-DF message is given in Appendix A1. The initial approach described in their paper used XML for the content description. However, O-MI and O-DF can also be represented using JSON [33], RDF and other formats that can be translated directly to and from XML. O-MI and O-DF may be used independently of each other. The role of O-MI/O-DF in the global context of IoT message transfer layers is illustrated in Figure 2, which is more detailed in [34].

O-DF is generic enough for representing any object and information that is needed for information exchange in the IoT. As shown in Appendices A1 and A2, the top-level “Objects” element may contain any number of “Object” sub-elements. Inside an “Object” element, users can include the object id with the description, as well as the main message content with the help of the “InfoItem” element. It is also possible to construct a hierarchical view of each object. The architecture is simple and generic enough, so that O-DF can be applied to ontology data communication (see also Section 3.3).

O-MI allows the read/write/cancel operation concerning the data that the message contains [28]. O-MI does not impose any message enclosing structure. In the O-MI convention, three types of operations should be followed while receiving an O-MI message: (i) “write” used for sending information updates to an O-MI node; (ii) “read” used for immediate retrieval of information and for placing subscriptions to an O-MI node; (iii) “cancel” for cancelling subscriptions before they expire.

As for the subscription mechanism, two types of subscription mechanisms are supported: (i) with callback address: the data are sent to the subscriber node using an O-MI response at the requested interval; the interval can be either “interval-based” or “event-based”; (ii) without call-back address: the data are memorized on the subscribed node as long as the subscription is valid. The subscriber can retrieve the memorized data (i.e., polling using subscription id) by issuing a new O-MI read query. Some application examples of O-MI and O-DF are explained in [30]. Such features were considered to be beneficial while integrating O-MI/O-DF in the semantic framework, which is detailed in the following section.

## 3. Semantic Framework for CL2M of Product-Service in IoT: Conceptual Architecture

For the purpose of responding to a holistic CL2M, which includes the notions of services, service lifecycle and actors, the Product-Service Lifecycle Ontology (PSLO) was modelled. The model was described in OWL2, using the Protégé ontology editor [35], while taking advantage of this language, demonstrated in [8,11,13,36,37,38]. Inside PSLO, several object property relations between product-service-actor objects were established in view of reusing the physical product ontology model provided by PLM SOM. The consistency check between the existing ontology and the extended part was achieved with the help of the Pellet [39] inference engine.

This section presents PSLO, as well as the framework of information sharing. Considering the detailed information of the PLM SOM ontology, which concerns the physical products’ lifecycle, readers are referred to [2,3]. Provided that IoT data shall be collected from different sources of data providers, which are heterogeneous and geographically distributed, it turned out that O-DF was a relevant message envelope.

### 3.1. Service Upper Ontology and Service Lifecycle Ontology

The main purpose of modelling an upper ontology is to provide a generic high-level model that might be extended into a more specific and detailed semantic model. The upper ontology allows the reusability of other parts, including generic rules and inference mechanisms inside the extended models. The upper ontology of PSLO integrates the characteristics of Product-Service Systems and requirements for service lifecycle management presented in different works [8,9,10,11,12,13,36,37,38,40,41].

As for the service lifecycle ontology, three key issues were considered: (i) ‘service lifecycle’ during the product use phase; (ii) EOL decision for material closing in the context of holistic CL2M; (iii) the possibility of representing actor engagement as of different stakeholders of PSS networks.

**Figure 3:**
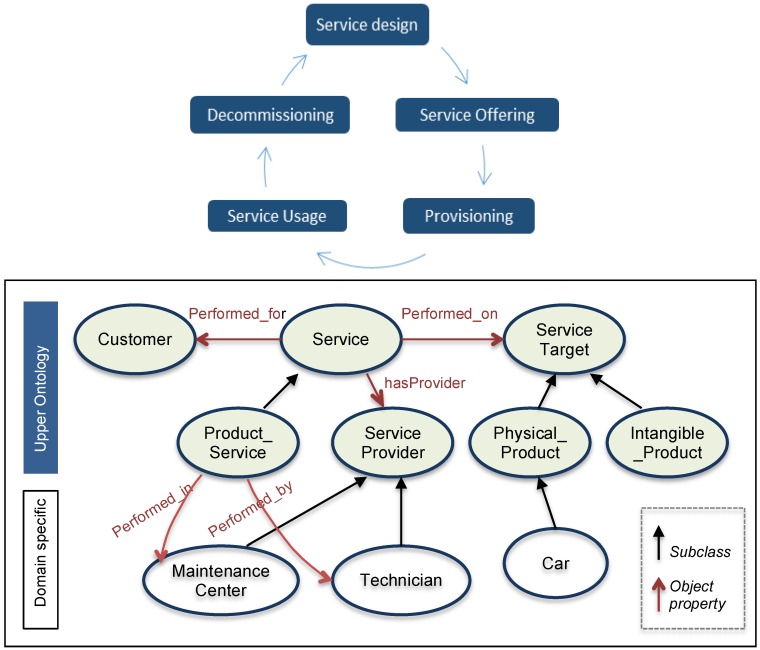
Service lifecycle phases and service upper ontology.

**Figure 4:**
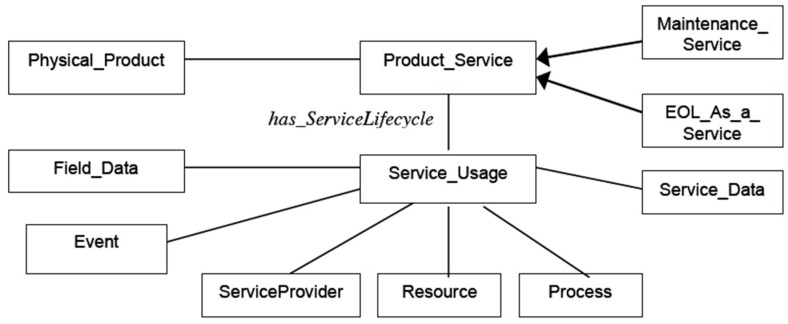
Product-service and the existing ontology of Physical_Product.

As far as the service lifecycle is concerned, the whole cycle is composed of: service design, service offering, provisioning, usage and decommissioning (top of Figure 3). The service upper ontology integrates four main elements: the definition of a service, a customer for whom the service is offered, the service provider who performs service operations and a service target on which specific service operations should be performed in case this concerns a product-service. Subsequently, the class ‘ServiceTarget’ which represent a service target can be further specialized into two subclasses: ‘Physical_Product’ for tangible services and ‘Intangible_Product’ for intangible services. The bottom part of Figure 3 shows the relation between the service upper ontology, which is represented by colour-filled elements, and the other domain-specific ontology elements. The domain-specific ontology is further detailed in the case study in Section 4. The notion of service illustrated in Section 4 deals with the ‘Service_Usage’, whose phase is overlapped with the physical products’ MOL phase (Figure 4).

### 3.2. Service and Product-Service

In PSLO, the notion of product-service is represented as a ‘Product_Service’ class, which is a sub-ontology of a more generic ‘service’ class. A generic service can be offered without associating a physical product, whereas a product-service should be created with an associated product object (i.e., link to the Physical_Product in Figure 4).

The product-service part is presented in Figure 4. The left-hand side elements, i.e., ‘Physical_Product’, ‘Field_Data’ and ‘Event’, are existing items in PLM SOM, whereas the other parts are newly-created elements. ‘Service_Usage’ has object properties, such as ‘service provider, ‘resource’ and ‘process’. As far as the associations with ‘Field_Data’ and ‘event’, their role is to link instance data of services to the PLM SOM ontology. The following description illustrates the reason for these semantic relations.

In PLM SOM, the product data were generated in terms of event data. Under the assumption that product-service data should take part in product lifecycle information, this notion should still be valid with the service data collection in PSLO. While maintaining such a semantic relation, our assumption was based on the fact that services or maintenance actions can be one of the main ‘events’ that indicate part changing or machine fault detection and repair. Depending on the product type, regular maintenance operations are even mandatory. Thanks to such maintenance histories, it is possible to retrieve richer product information while measuring the rate of system failure and/or the functional satisfaction level of product parts, for instance.

‘Maintenance_Service’ or ‘End-Of-Life_As_a_Service’ is a type of specialized ‘Product_Service’. EOL operations can contain attributes that indicate initial products and post-products resulting from refurbishing or remanufacturing processes according to an EOL decision. Such data can be useful with information search about pre-lifecycle during the other phase of products lifecycle, e.g., the product redesign in BOL.

**Figure 5:**
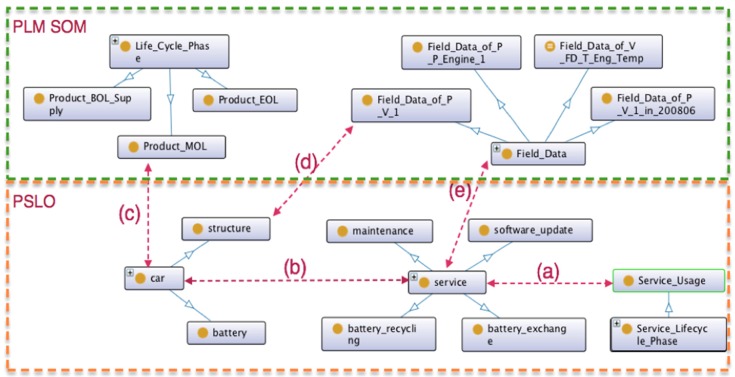
Global view of the physical product and product-service ontology model.

A part of PSLO is demonstrated in Figure 5 for the purpose of showing the relationship between physical product, service and respective lifecycle phase information. Hereby, we will use the word ‘service’ designating a product-service in general for the sake of simplicity. Figure 5a represents the link from Service_Usage (upper ontology) to the service instance (in a domain-specific ontology). Figure 5b represents the internal object property relation between the physical product ‘car’ and ‘service’, which will be detailed in Section 4. As far as Figure 5c is concerned, the car object is in the MOL phase. The maintenance or event data can be represented as filed data Figure 5e, which are the counter part of the relation between a specific field data type and some parts of physical products Figure 5d.

### 3.3. Semantic Info-Node Agent Architecture in an Executable Instance of Framework

PSLO was created using Protégé 5.0 and written in OWL2 RDF/XML serialization. Using the ontology editor and SPARQL query window, users can verify inferred instances or results provided by a rule engine. In addition to the publication of the ontology, the ultimate goal is to provide a framework for PLSO knowledge sharing in an IoT-enabled environment. Consequently, the ontology framework is instantiated on OpenRDF Sesame [42]. Sesame is an open source Java framework for processing RDF data. The framework offers a wide range of tools to developers to leverage the power of RDF and related standards, including parsing, storing, inference and querying of/over semantic data. It offers an easy-to-use API that can be connected to all leading RDF storage solutions. It allows us to connect with SPARQL endpoints and create applications that leverage the power of linked data and the Semantic Web.

Sesame offers two out-of-the-box RDF databases, i.e., the in-memory store and the native store, which are endorsed by many third party storage solutions. Sesame fully supports the SPARQL 1.1 query and update languages for expressive querying and offers transparent access to remote RDF repositories using the same API as for local access. Sesame supports all mainstream RDF file formats, including RDF/XML, Turtle and JSON-Linked Data (JSON-LD).

The following list shows some available third-party database solutions that implement the Sesame APIs.

- CumulusRDF is an RDF store on a cloud-based architecture, fully compatible with the Sesame APIs. CumulusRDF provides a REST-based API with create, read, update, delete (CRUD) operations to manage RDF data. The current version uses Apache Cassandra as the storage backend.
- Sesame Adapter for Oracle Database: an Oracle-developed adapter component for accessing Oracle Semantic Technologies via the Sesame APIs.
- Strabo is a geospatial RDF triple store based on Sesame.
- Virtuoso Sesame Provider is an OpenLink-developed component that allows accessing an OpenLink Virtuoso triple store via the Sesame APIs.

**Figure 6:**
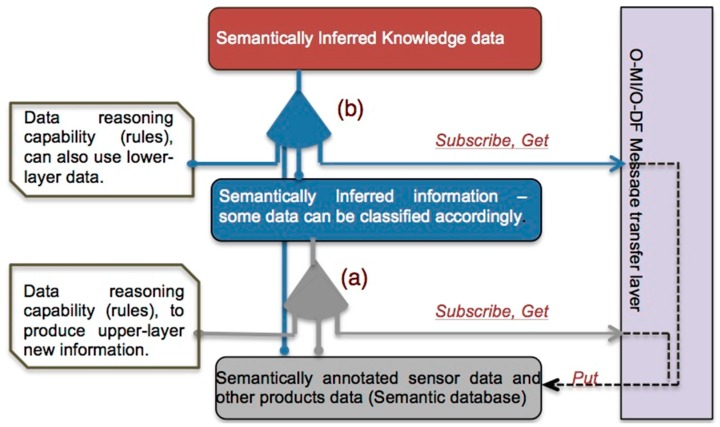
Semantic info-node agent architecture.

After that PSLO is transferred to Sesame, the O-MI/O-DF node agent, Semantic Info-Node Agent (SINA), is created while providing additional features required to access and update Sesame databases. Figure 6 illustrates a simplified view of the SINA architecture.

The agent behavior is based on a deliberative and communicative agent architecture [43]. In terms of technical implementation, SINA is a Java thread object that is composed of a message box, internal state, Sesame database access functions and SPARQL and the SPARQL Inferencing Notation (SPIN) rule scripts, if applicable. One of the agent internal properties includes the namespace definition referring to its ontology model. A particular namespace makes a unit SINA specific to a certain domain of ontology. The agent messages are described in the O-DF message syntax. Appendix A(b) shows an example of a data transfer message relative to SINA. The enclosed block of O-DF InfoItem is parsed by the agent. Within an O-DF object “sina-rdf”, users can specify InfoItem elements: for example, for the agent identifier (InfoItem = “agent-space”) or for the data to write (InfoItem = “rdf-content”). The “rdf-content” element is the RDF graph data to be communicated for data writing.

Thanks to an agent’s internal rule description and inference engine, the agent is capable of generating new information (Figure 6a). The newly integrated information can recursively contribute to another rule description (at an upper-level SINA), subsequently creating other types of knowledge (Figure 6b). Such a framework allows one to continually collect data, infer and share inferred information through IoT message transfer. In comparison with the conventional IoT data messaging in which the transfer of hardware sensor data is the main objective, the framework makes it possible to share the inferred information as a kind of abstract sensor data. For the moment, a simple O-DF parser was developed for data collection, update and retrieval, without considering the full range implementation, which includes O-MI node functions. However, if the framework is connected with a full O-MI server node, it will be possible that other agents or IoT APIs can subscribe to collecting the SINA node’s knowledge data. To summarize, the framework helps extend the notion of IoT from physical sensor data towards an abstract level of logical data [44].

## 4. Electric Car and Battery Lifecycle: Case Study on Domain-Specific Ontology Creation

This section demonstrates the use case of PSLO in the context of electric car and battery lifecycle information management. The above-presented ontology concept was applied to a specific product-service type, i.e., electric car service, integrating actors and service details, as well as their instance data.

The choice of the case study was mainly derived from the importance of service lifecycle regarding this specific product. As discussed in [38], the needs for an approach based on Product-Service System (PSS) management become more important than before, especially in new or evolving markets, such as the electric car industry. Particularly, the management of product maintenance and service records play an important role, since they provide the information of data generation time (i.e., event) about the notice of failure or worn-out rate during a certain period of time. As mentioned by the same authors, from a consumer perspective, the electric car is substantially different from the traditional car. Although it meets a similar need to conventional cars, the electric car has a number of innovative features. The service component is particularly important as one of the relevant factors in decisions to purchase electric cars. That is the main reason that we were interested in applying our methodology to an electric car case study.

For that purpose, the upper ontology was specialized into a domain-specific ontology of electric car and related services. The electric car product subsystems presented in [45] were the main reference of our domain-specific model. Among four subsystems of the electric car, i.e., vehicle sub-system, electronics on-board sub-system, infrastructure sub-system and energy sub-system, this paper focuses more on the ontology model of “vehicle sub-system” and “energy sub-system”: the car with the other structure for the former and the electric battery for the latter subject. In the following subsection, the ontology model is detailed.

### 4.1. Domain-Specific Ontology Model

**Figure 7:**
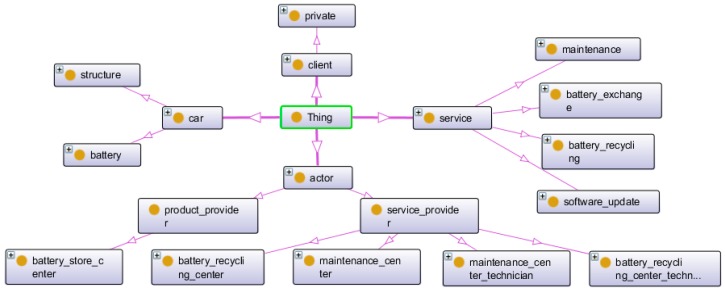
Overview of the domain-specific ontology of EV service.

Figure 7 represents a semantic model for an electric car and related service classes modelled using Protégé. It includes four main classes: actors, cars, clients and services. For the sake of understanding the model, we will briefly describe the four main classes actors, cars, clients and services and their principal relations (subclasses and object properties in Protégé).

For the sake of understanding the model, we will briefly describe the four main classes actors, cars, clients and services and their principal relations (subclasses and object properties in Protégé).

#### 4.1.1. Car and Client Classes

**Figure 8:**
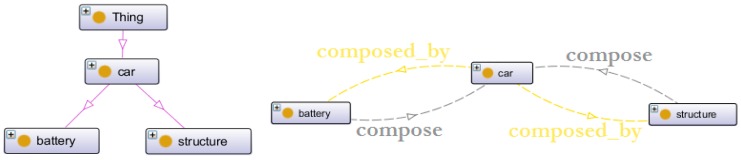
Overview of the car class and object properties.

Figure 8 depicts the car class has two subclasses: battery and structure. It has been decided to separate the battery from the drive unit due to the fact that, with an electric car, they are independent of one another. Moreover, they may have different lifespans, and most of the services focus on only one part.

Creating further interaction between classes requires object properties. The objects’ properties link one class to another according to a semantic meaning given with the relation. Concerning the car class, there are two object properties defined between battery and structure.

**Figure 9:**
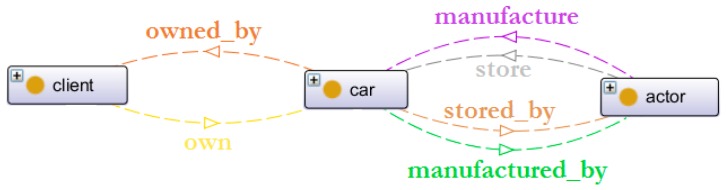
Object properties linking the actors, cars and clients.

The car is manufactured (battery and structure), then stored in an actor’s business location (i.e., product-manufacturer centre or service-provider centre), and finally, the client owns the car after purchasing it. The corresponding object properties are illustrated on Figure 9.

#### 4.1.2. Actor Class

**Figure 10:**
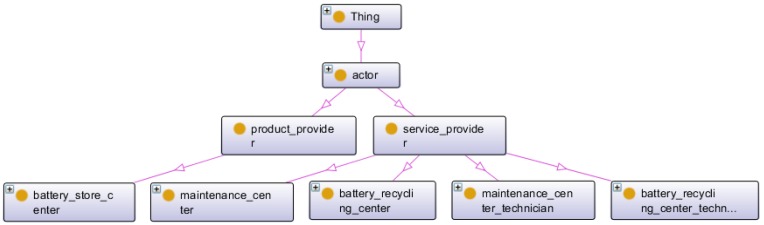
Overview of the actor environment.

The actor class includes product-providers (providing the battery and the structure of the car to the client) and service-providers (providing the services). The service-provider class is divided into four sub-classes: two centre classes (where the service is performed) and two technician classes (by whom the service is performed). Figure 10 illustrates the actor class and semantic relations with other classes.

**Figure 11:**
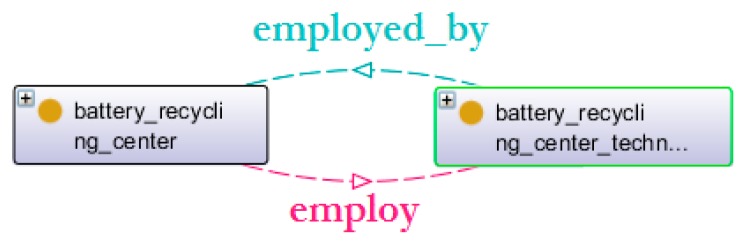
Object properties of the actor environment.

There are two objects properties linking the centres with the technicians (Figure 11): employ and employed-by.

The actor environment includes individuals of maintenance centres, battery recycling centres, technicians for maintenances and technicians for battery recycling.

#### 4.1.3. Electric Car Services Class and Relations

**Figure 12:**
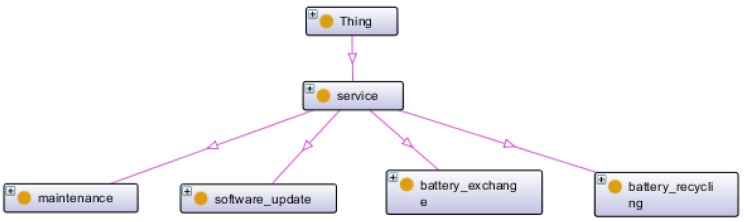
Overview of the service and its subtypes.

Here, as for the case study example, we created four types of specific services that are shown in Figure 12.

- Maintenance: Regular maintenance performed by a technician. We assume a maintenance service is performed every year from the date of purchase of the car during the service contract period.
- Battery exchange: Battery exchange, i.e., swapping, is an alternative to recharging and concerns swapping a drained or nearly drained battery with a fully-charged battery, which is operated in battery exchange stations.
- Battery recycling: This service concerns the recycling of old batteries either after swapping or after complete change.
- Software update: A software update is offered to the client, at a relevant moment, by the car manufacturer. Since it is not mandatory, the client can choose whether to install it or not.

**Figure 13:**
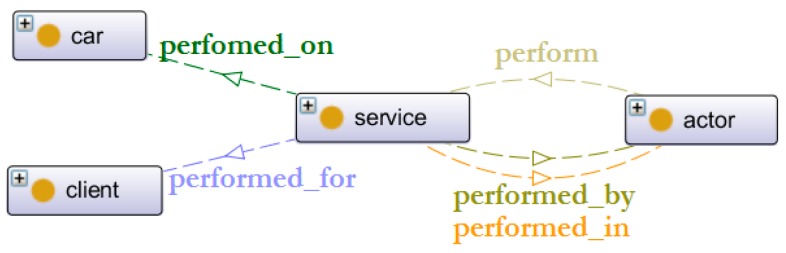
Objects properties of the service.

Figure 13 details five object properties which are assigned to a service object, which are described below:

- perform: Which service is performed (e.g., a maintenance)?
- performed_by: By whom is the service performed (e.g., a technician)?
- performed_for: For whom is the service performed (e.g., a private client)?
- performed_in: Where is the service location (e.g., a maintenance centre)?
- performed_on: On which product is the service performed (e.g., a battery or a structure)?

In this case study, the class ontology and individuals are created manually with the help of the Protégé editor. Afterward, the OWL ontology is transferred to the Sesame environment for the purpose of being used as a SINA repository. Using IoT message passing, the instance data can be progressively incremented, which was tested with some selected message passing, e.g., maintenance records or battery wear level. As for the data retrieval, it is also possible to launch SPARQL queries directly into the ontology base or to verify the data instances with the help of the Protégé SPARQL query tab, which is discussed in the following subsection. 

### 4.2. Data Retrieval Example

The following SPARQL query is used for the purpose of retrieving all of the services of a client, whose name is Alexandre Grozel:


SELECT ?service_performed ?service_date
WHERE{
?who a tes:private.
 ?who tes:private_name ?private_name.
 ?service_performed tes:performed_for ?who.
 ?service_performed tes:service_date ?service_date.
 FILTER (?private_name="alexandre grozel"^^xsd:string)
}
ORDER BY ?service_date


**Figure 14:**
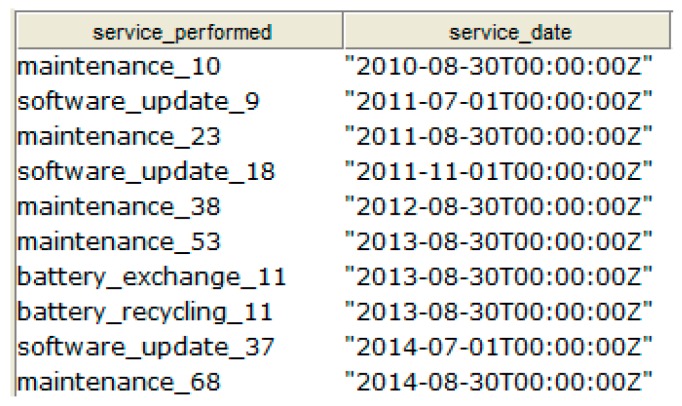
Services used by the client “Alexandre Grozel”.

The output searched by Protégé is displayed in Figure 14.

Provided that the services are identified, the following query is used for the purpose of finding the characteristics of the “battery_exchange_11” service.



SELECT ?service_performed ?service_date ?battery_exchange_old_battery ?battery_exchange_new_battery ?center ?technician
WHERE{
?service_performed a tes:battery_exchange.
?service_performed tes:performed_for ?private.
?private tes:private_name ?private_name.
?service_performed tes:service_date ?service_date.
?service_performed tes:battery_exchange_old_battery ?battery_exchange_old_battery.
?service_performed tes:battery_exchange_new_battery ?battery_exchange_new_battery.
?service_performed tes:performed_by ?technician.
?service_performed tes:performed_in ?center.
FILTER (?private_name="alexandre grozel"^^xsd:string)
}


**Figure 15:**
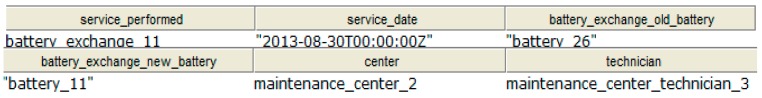
Output produced by Protégé according to the query description.

The output produced by Protégé is given in Figure 15.

The key point here is that data providers of retrieved information might be heterogeneous in terms of time, location and their roles. Each actor or service business can generate a spot of data and send it to the SINA O-DF endpoint, so that the content information can be integrated into PSLO RDF databases. The inferred result can also be referenced or combined with any other information on customers or car design, which are often selected as factors to be examined in view of product redesign or new service creation.

**Figure 16:**
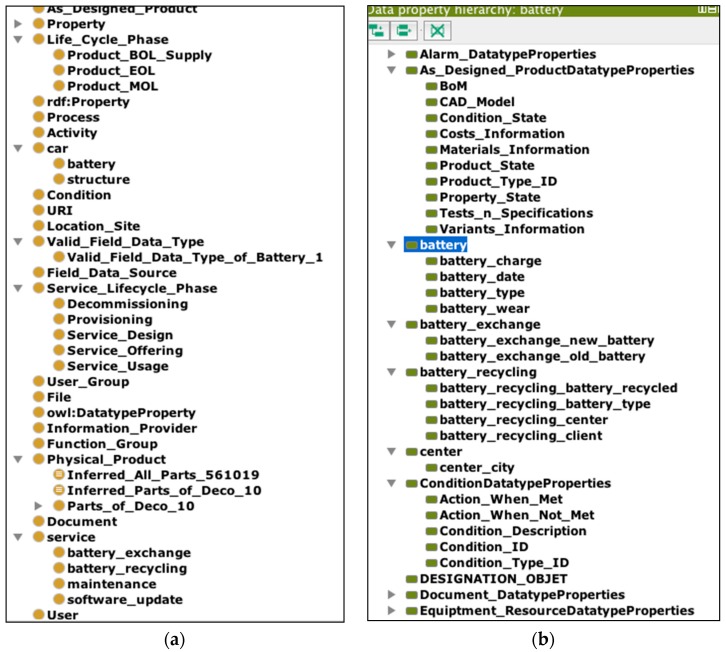
PSLO with the domain-specific ontology of the electric car service.

Figure 16 illustrates another view of the whole PSLO ontology: (a) illustrates PSLO composed of the physical product ontology with services and the service lifecycle; (b) shows some examples of the data properties used in the upper ontology, as well as the domain-specific ontology of product-service, which are currently integrated.

## 5. Discussion

In this section, we evaluate the results while answering the following questions:

- How does the framework enhance information extraction in the context of the product-service lifecycle?
- What are the benefits from the extension of PLM SOM in view of modelling integrated PSLO?
- What are the limitations to be improved?

### 5.1. How Does the Framework Enhance Information Extraction in the Context of Product-Service Lifecycle?

For the sake of demonstration, here is a simple example of ontological rules that show how inference-based information extraction can occur in PSLO. The example situation happens at a car manufacturer’s site. According to a production strategy, a car company wants to find the functional weakness or point of improvement of a battery type. In order to better service customers in the near future, the company is planning to accompany a new service type integrated with the car on which the new battery will be installed. In the context of the service design and promotion, the manufacturer wants to establish the potential customer group by finding all cars on which the old batteries are installed and used and then to find current customers of the cars so that they are informed of the new service by direct communication. 

A series of processes for information extraction should be achieved on the PSLO ontology, following the sub-procedures presented below.

- Define a new inferred class ‘Battery_Drawback’: as for the category of batteries to examine, we assume that the manufacturer wants to retrieve all of the batteries manufactured before a given date (24 September 2011, for example) for which the battery wear level has been marked as below 70 (which might be an imaginary thread value for functional check).
- Find all of the cars on which those batteries were installed.
- Find all of the customers who had used, or have been using, the car, as well as the service offering associated with the car, if applicable.
- Define a new inferred class ‘Future_Contact’ as a potential service user group, while categorizing those customers into the future group of service clients, and save them in the knowledge base.

**Figure 17:**
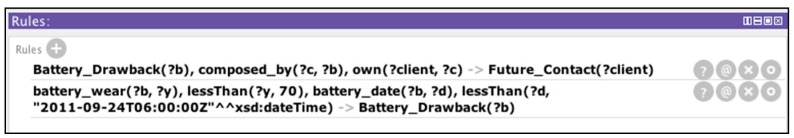
Protégé rule tab containing the inference queries.

Such an idea was mainly inspired from the product function improvement described in [45]. Figure 17 shows rules modelled in the Protégé rule tab that correspond to the above-mentioned processes.

It is also possible to find other interesting data, which are also listed below.

- Find all of the services associated with this battery
- Find product the design documents of the battery: for the purpose of retrieving the design information during the beginning-of-life of the product

**Figure 18:**
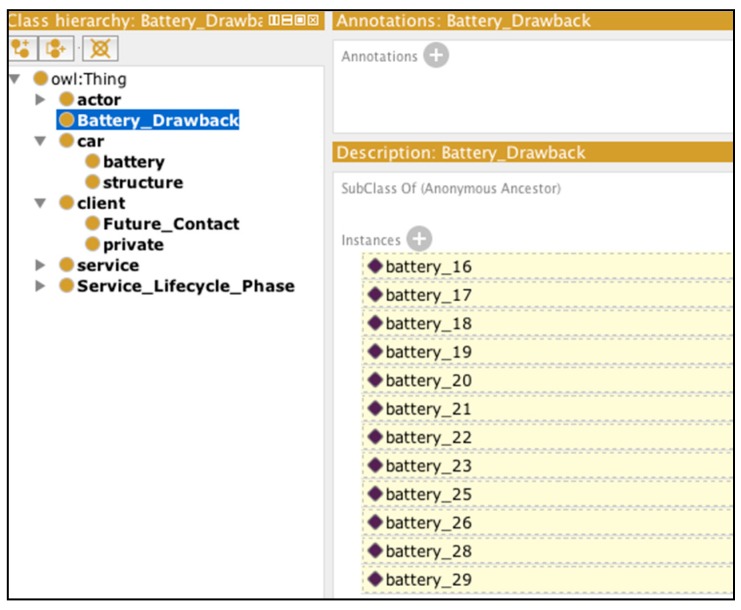
‘Battery_Drawback’ inference example.

**Figure 19:**
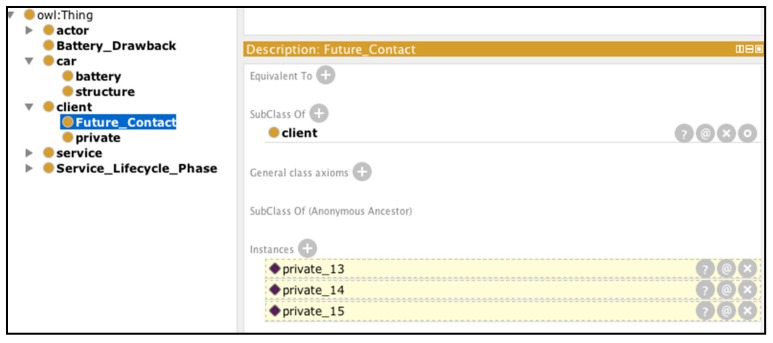
Inferred knowledge on the customer to contact in the near future.

We used the Pellet [39] reasoner installed on Protégé, which returns the results as illustrated in Figure 18 and Figure 19. The reasoning result is different from a query result in that the inference engine is continuously running in real time. As soon as new data are entered into the system, new results are retrieved. Thanks to the rules, the Battery_Drawback class now includes inferred instances according to the data available. If the data are incrementally added, the inferred instances change accordingly (Figure 18).

As we can observe in Figure 18, new information is visible under the form of Protégé inferred instances being marked in yellow-filled lines. Similarly, Figure 19 displays the inferred client instances, which are extracted recursively based on the results of Battery_Drawback.

**Figure 20:**
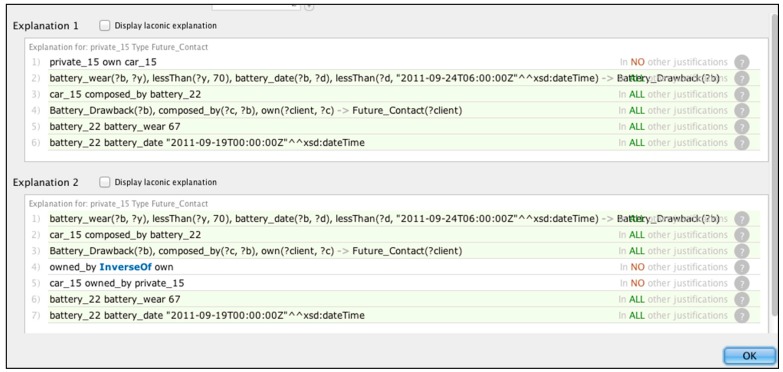
Tracking the reason for the inferred results.

One of the useful functions with the inference procedure is the possibility to learn the reasoning processes behind the extracted information. Figure 20 shows the explanation of reasoning about the ‘private_15’ client, i.e., the battery wear level of that client was under the given level value (less than 70), whereas the manufacturing data of that car was before the given date constraint. Concerning the inferred knowledge, Protégé allows us to verify the reason of the inference data. For example, by clicking the customer ‘private_15’ element on the ‘?’ button, users can see the explanation of the inference processes.

### 5.2. What Are the Benefits from the Extension of PLM SOM in View of Modelling Integrated PSLO?

Firstly, the existing PLM SOM integrates not only a class-level ontology model, but also instance data, which were already validated by the authors [2,3] with the help of an appropriate inference engine. Therefore, the validity of ontology model, as well as its instance data was checked for logical consistency, upon which the other part of the PSLO model could be safely built.

Secondly, the origin of the product data in PLM SOM was collected from a physical product sensor in the context of the former project. Therefore, the data semantics at the level of product information represented well the real-world product model. Even though the product category was not an electric car, we could build a reasonable electric car model while adding fictive part objects, which were specific to the case study, e.g., an electric battery object. Subsequently, while generating a specific ontology driven from the upper ontology, the extended model could remain homogeneous in terms of product type at its lower level object semantics.

Finally, the existing PLM SOM was rather a vertically-closed ontology model considering only the physical product and the MOL product data, which was one of the shortcomings of the existing approaches that we discussed in Section 2. Consequently, throughout the research, we wanted to show how to break such vertical silos thanks to the integration of the product service ontology while including actors and services. The horizontal coordination was achieved while implementing semantic links between ‘car’ and ‘Product_MOL’ ((c) on Figure 5), ‘service’ and ‘Field_Data’ (f), or field data collection (‘Field_Data_of_P_P_Engine’) from one of the structure parts (d), for instance, as illustrated on Section 3.3, Figure 5.

### 5.3. What Are the Limitations to Be Improved?

Despite the demonstrated interests, there are still open issues that must be solved in the near future so that the development of the product-service ontology may become mature as soon as possible. Some discussions in [13,40] are summarized below:

- Semantics for each term should be defined properly to avoid ambiguity between stakeholders, including not only researchers, but also industries: For the moment, the community demonstrated very few efforts in standardization. The same authors envision that the terminologies proposed within the domain of PS and PSS will increase exponentially in the forthcoming years. A common vocabulary in PSS should be established rapidly in order for a semantic approach to be widely accepted as a sharable way of dealing with cross-domain product-service lifecycle management.
- A common software platform and methodology needs to be developed to update the ontology in a progressive way. Current IoT-related open platforms and data communication standards might be valuable to enable such a common platform.
- A PSS ontology should be intuitive so that it could be easily and appropriately implemented in industries: we need joint work efforts with the domain specialists and research results from the PSS design and methodologies: A more flexible and modular approach will fit better rather than sticking to a conventional approach to a hard-wired closed platform.

As we described in Section 2.2, there are three views of ‘ontologies’ that are commonly accepted: as models of reality; as models of information; and compendia of controlled terms. Our PSLO framework provides mainly the models of the information of the product-service lifecycle and, most importantly, the compendia of controlled terms, which clearly annotate the world of product-services, data and actors. From our perspectives, the PSLO ontology will be useful with the definition of the common vocabulary of product-services and PSS.

## 6. Conclusions

In this paper, we presented a conceptual framework for closed loop lifecycle knowledge sharing, which is enabled by an ontology and semantic modelling approach, on the one hand, and standard IoT message transfer, on the other hand. The semantic modelling approach and IoT standard both play an important role in the context of CL2M achieving complementary objectives. IoT standards at the message layer are important for describing a data transfer scheme, whereas an ontology language, such as OWL2, allows us to model the domain knowledge, as well as to infer knowledge.

The case study demonstrated in this paper is a fictive example. However, the provided framework can be used in a real-world case once the sensor data can be collected directly from products during their lifecycle. The semantic database is implemented in Sesame APIs for further improvement and extension. In the following subsections, the contribution of the work, research findings, as well as the plan for future work are discussed.

### 6.1. Contribution of Our Approach to PLM and IoT and How It Complements Traditional Approaches

The main contribution of the conceptual framework is that it can act as an enabler of a synergy effect driven from both domains, PLM and open IoT systems. The ontology model is the supporting backbone of IoT data while providing PLM objectives with the inference capability.

The traditional PLM focused on the physical product data collection within a closed silo of information management. In that regard, product-generated data are collected according to a predefined objective. At least two limitations can be recognized: (i) firstly, the management purpose of product lifecycle data can hardly be evolving in line with the consumer preferences or social context, which are highly volatile and continuously evolving in today’s ICT environment; (ii) secondly, it is very difficult or even impossible to establish horizontal data sharing among existing information management systems.

In our experience, a semantic approach provides a promising solution to breaking such barriers aiming to achieve knowledge management with more evolution and flexibility. If the framework meets a real-world working example, the collected data might include heterogeneous information in terms of semantic level and data providers. In other words, the data might include physical sensor-generated data, as well as logically-inferred data and/or human-observed information. The condition to satisfy is that all data should be annotated according to a given language rule described in RDF, and enveloped within the O-DF format.

Referring to the discussion in Section 2.3, the purpose of using ontology in this work is to take advantage of the first and second points: semantic interoperability to support the integration of data between different sources. IoT data usually originate from a device or a human and refer to the attributes of a phenomenon or an entity in the physical world. Thanks to the interoperability offered by the ontology, the data can be combined with other data to create different abstractions of the environment or they can be integrated into the data processing chain.

### 6.2. Research Findings

Based on our experience and the obtained results, here are some findings summarized below.

(1) The use of RDF/XML and OWL2 as the data content in O-MI/O-DF allows broadening the scope of transferred information. The traditional sensor data transfer mainly concerned the product state information assigned to attributes, e.g., temperature or state, or maintenance data, whereas now, the IoT data can even include the object properties (i.e., semantic relations) between IoT or even rule contents. Some such examples include: the battery “was detached from the car”, an owner “purchased a car”, and so on. All information can be exchanged using the same message envelope. It turned out that the semantic framework gives a chance to enlarge the knowledge content available for enhanced PLM.
(2) The horizontal linking of data silos is realized while relevantly establishing object level relations. Not only breaking the vertical silos, our approach makes it possible to achieve flexible evolution in the whole PSS. This is due to the fact that the RDF data can transfer the new class before gathering real IoT data or, inversely, new types of data can be transferred without specifically mentioning the category. The knowledge inference is nonetheless possible by relevantly using semantic rules in OWL2 and SPARQL.
(3) As far as the type of databases is concerned, an RDF database provides a convenient environment for the purpose of populating semantically-annotated IoT data. Otherwise, an additional translation step is required in order to convert the semantic annotation into other serialization formats usually accepted by O-MI/O-DF nodes. Another possible case concerns the IoT data written in XML Schema, whereas the product-service ontology should receive those data for knowledge inference. In this case, the necessary step requires data conversion from usual XML Schema format data into the ontology-fitted RDF version.

### 6.3. What Is the Near Future Development?

The works in progress can be summarized into two types of activity: (i) improving technical aspects of the framework and message communication layer; (ii) providing some rules at the level of product-service upper ontology which might be applicable to the domain specific ontology.

On the one hand, the technical aspects include the improvement of the O-MI/O-DF parsing function while accepting other types of script language, such as JSON or JSON-LD. For the moment, the SINA agent takes exclusively the ‘InfoItem’ content in RDF/XML format while parsing the internal block of an O-DF. This might be a restriction that hinders a wide adoption of the approach. Another issue concerns the improvement of access functions on the Sesame repository. At the current stage, a predefined PSLO ontology was directly transferred to the RDF database without filtering. However, it might be more useful to provide several ontology domains that can be accessible by several SINA agents.

On the other hand, more rule examples are being researched in order to enhance the knowledge sharing between the product data and service MOL data, which was addressed through a methodological approach in [8,46]. The work presented in [47] gives a good insight into a generic framework for that purpose. In their work, the event semantic link was studied and reasoning rules for the event semantic link network were suggested according to a generic definition of situations. Such a rule set can allow intelligent reasoning of dynamic knowledge while tracing the evolution of event generation.

### 6.4. Concluding Remarks

For the purpose of achieving a holistic approach to CL2M, the explicit integration of service and maintenance activities is an important precondition of information extraction. Regarding this aspect, we suggested an extended semantic model of product-service lifecycle ontology, which includes services with physical products. In this paper, the methodology was applied to a specific case study. However, the approach can be generalized in a more global context taking into account other types of products and data. As we mentioned in Section 2.2, understanding a specific ontology requires first of all understanding the context of the modelling objective and its expected use. The focus of our product-service ontology was initially set with the aim of enhancing the information and knowledge sharing among different phases of the lifecycle. The result can be applied to another context in case the purpose of the ontology is approved as a similar context. The expected result will then materialize, enabling product-service information exploitation and knowledge sharing in an open environment.

## Figures and Tables

**Table 1:** Summary of Section 2.1. PSS, Product-Service System.

	Definition	Approach or Methodology to PSS
[8]	Product service: an integrated combination of tangible products and intangible services	Give the capability to offer a flexible value proposition to customers throughout the lifetime of a product
	PSS: social systems that enhance social and economic value received by each actor in the network through the mutual provision of a PS	
[9]	PSS: a marketable set of products and services capable of jointly fulfilling a user’s need	Theoretical framework for PSS composed of five key elements: 1. Products, services and their combinations; 2. Services at the point of sales; 3. Different concepts of product use; 4. Maintenance services; 5. Revalorisation services
[10]	Product-service: composed proposition of tangible products and intangible services designed to optimize the product use and increase the value	Smart maintenance service for strategic progress of PSS from product-oriented to a service-oriented lifecycle
[11]	PSS: definition based on [9] referenced	Framework integrating technical product with non-physical service by an automated design processes
[12]	PSS: a system of products, services, supporting networks and infrastructures that are designed to be competitive, satisfy customer needs and have a lower environmental impact than traditional business models, according to [9]	Classification of PSS model based on three dimensions: Product-oriented services, Use-oriented services, Result-oriented services
[13]	PSS: an integrated product and service offering that delivers value in use to the customer	Several methodologies presented including Fast-Track Total Care design process, Heterogeneous IPS concept modelling, and Dimensions of PSS Design

## Abbreviations

The following abbreviations are used in this manuscript:

CL2M	Closed-Loop Lifecycle Management
PSS	Product-Service System
IoT	Internet of Things
URI	Uniform Resource Identifier
PLM	Product Lifecycle Management
BOL	Beginning of Life
MOL	Middle of Life
EOL	End of Life
O-MI	Open Messaging Interface
O-DF	Open Data Format
SPARQL	SPARQL (or Simple) Protocol And RDF Query Language
SPIN	SPARQL Inferencing Notation

## Appendix A. Example O-DF Message Structure

1. O-MI and O-DF message example: The following example shows how to push information using O-MI, which contains O-DF message content. The dotted box area contains the O-DF message, whereas the outside part concerns the O-MI message syntax.

2. O-DF, which includes RDF message content: information about an instance of the battery. The dotted box contains the Semantic InfoNode Agent (SINA)-specific message.
